# Pyrogallic Acid in Phagædenic Ulceration

**Published:** 1882-02

**Authors:** 


### Pyrogallic Acid in Phagædenic Ulceration.

M. Vidal, after using pyrogallic acid with care in the treatment of psoriasis, has tried a salve with good effect to heal phagædenic ulcers and to cicatrize chancres. He applied it to a phagædenic ulcer daily for three days, and states that the pain caused is only moderate, and lasts but from eight to ten minutes. The formula he recommends is acid pyrogallique 20 grammes and axonge or vaseline 100 grammes.—*Bull. Soc. de Therap.*

